# The Emotional and Attentional Impact of Exposure to One's Own Body in Bulimia Nervosa: A Physiological View

**DOI:** 10.1371/journal.pone.0102595

**Published:** 2014-07-18

**Authors:** Blanca Ortega-Roldán, Sonia Rodríguez-Ruiz, Pandelis Perakakis, M. Carmen Fernández-Santaella, Jaime Vila

**Affiliations:** 1 Department of Personality, Evaluation and Psychological Treatment, University of Granada, Granada, Spain; 2 Laboratory of Experimental Economics, Department of Economics, University Jaume I, Castellón, Spain; INSERM U894, Centre de Psychiatrie et Neurosciences, Hopital Sainte-Anne and Université Paris 5, France

## Abstract

**Background:**

Body dissatisfaction is the most relevant body image disturbance in bulimia nervosa (BN). Research has shown that viewing one's own body evokes negative thoughts and emotions in individuals with BN. However, the psychophysiological mechanisms involved in this negative reaction have not yet been clearly established. Our aim was to examine the emotional and attentional processes that are activated when patients with BN view their own bodies.

**Method:**

We examined the effects of viewing a video of one's own body on the physiological (eye-blink startle, cardiac defense, and skin conductance) and subjective (pleasure, arousal, and control ratings) responses elicited by a burst of 110 dB white noise of 500 ms duration. The participants were 30 women with BN and 30 healthy control women. The experimental task consisted of two consecutive and counterbalanced presentations of the auditory stimulus preceded, alternatively, by a video of the participant's own body versus no such video.

**Results:**

The results showed that, when viewing their own bodies, women with BN experienced (a) greater inhibition of the startle reflex, (b) greater cardiac acceleration in the first component of the defense reaction, (c) greater skin conductance response, and (d) less subjective pleasure and control combined with greater arousal, compared with the control participants.

**Conclusion:**

Our findings suggest that, for women with BN, peripheral-physiological responses to self-images are dominated by attentional processes, which provoke an immobility reaction caused by a dysfunctional negative response to their own body.

## Introduction

One's own body is a complex stimulus that may generate dysfunctional emotional and attentional responses in people with eating disorders such as bulimia nervosa (BN). Body dissatisfaction, defined as a cognitive-emotional distortion related to self-image [1], [2], is the most common among these dysfunctional responses. Dissatisfaction and preoccupation with body shape and weight influences the onset of BN and has been suggested as a primary symptom in maintaining the disorder [3]. Furthermore, the persistence of body dissatisfaction after treatment of eating disorders is associated with relapse in patients with BN [4]. The majority of therapeutic interventions aimed at reducing body dissatisfaction use exposure techniques to attenuate negative emotions associated with the patient's own body [5]–[9]. These interventions are based on the hypothesis that patients with BN perceive their body as an unpleasant stimulus that elicits aversive emotional states. Empirical evidence in support of this idea comes from studies reporting that patients with BN experience increases in negative thoughts and emotions while viewing or imagining their own body [10]–[13]. In addition, patients' physiological responses to self-images also resemble those evoked by unpleasant stimuli: increased heart rate [14], [15], increased skin conductance recovery time [15], and high activity in the right temporal lobe [16].

On the other hand, increasing evidence suggests that viewing one's own body also involves significant attentional processing. First, body checking, an important component in body image disturbances, which is conceptualized as the repeated monitoring of body shape or weight [17], leads to attentional biases towards body-related cues; e.g., individuals asked to focus on and inspect different body areas are faster at detecting body-related cues compared with neutral stimuli and report more body dissatisfaction [18], [19]. Second, eye-tracking studies have shown that, while viewing pictures of their own body, people with eating disorder-related symptoms demonstrate increased pupillary dilation and decreased blink rate [20], both responses considered indexes of increased attention and concentration. Third, eye blink inhibition, together with decreased activation of the facial musculature, suggesting enhanced attentional processing, was observed in response to self-image exposure in healthy young women [21].

Given the importance that body dissatisfaction plays in the treatment of BN [1], [3], [22], [23], elucidating the precise relationship between its attentional and emotional components will contribute not only to an improved theoretical understanding, but also to the development of efficient therapeutic strategies. In this study, we used the startle-defense response paradigm [24], [25] to examine the attentional and emotional mechanisms activated while viewing a video with a rotating picture of one's own body. Two groups of participants were compared: a group of patients with bulimia nervosa (the BN group) and a control group of healthy individuals (the HC group). The startle-defense paradigm was chosen because it allows to examine both, the attentional and the emotional activation by simultaneously eliciting motor (startle) and autonomic (cardiac defense) responses.

The startle reflex is a motor response characterized by a quick closing of the eyes accompanied by a stiffening of the head, dorsal neck, body wall, and limbs [26], [27]. It is elicited by aversive stimulation —usually, unexpected loud noises of short duration—. The magnitude of the reflex is potentiated when the noise is preceded by a highly arousing and unpleasant stimulus [28]. This potentiation is explained by the priming effect that the preceding stimulus exerts on the neural circuit that controls defense reactions [29]–[31]. In contrast, inhibition of the startle reflex by prior presentation of highly arousing, pleasant stimuli is associated with the activation of the appetitive motivational system [32]. Several studies, however, provide empirical evidence suggesting that startle inhibition may also occur when attention is directed away from the modality of the startle probe, especially if attention is directed to a stimulus of high cognitive load [33]–[35].

The cardiac defense response refers to the pattern of heart rate changes that occur in reaction to an aversive stimulus —usually, a sudden short loud burst of white noise— and is characterized by two acceleration-deceleration components that occur during the 80 seconds after stimulus onset: (a) an initial heart rate acceleration with a maximum peak at 2–3 seconds followed by a sudden deceleration; and (b) a second, more gradual acceleration with a maximum peak approximately 35 seconds, followed by another more gradual deceleration [25], [36]. However, this complete response pattern tends rapidly to decrease in amplitude and even disappear upon stimulus repetition [25], [37]. As in the case of the startle reflex, the cardiac defense response can be modulated by attentional and emotional factors. The first acceleration-deceleration component, mediated by parasympathetic cardiac control, is affected by attentional factors and is interpreted in terms of passive defense (i.e., the interruption of ongoing activity and increased attentive response) [25], [38]. In comparison, the second acceleration-deceleration, mediated simultaneously by sympathetic and parasympathetic cardiac controls working reciprocally, is affected by emotional factors and is interpreted in terms of active defense (i.e., the preparation for fight or flight) [39].

Based on the above, an *emotional* dysfunctional response to one's own body (perceived as an aversive stimulus) would predict a specific pattern for the modulation of the eye-blink startle and the cardiac defense: simultaneous potentiation of the eye-blink [40], [41] and of the second acceleration-deceleration component of the cardiac defense [25]. Conversely, an *attentional* dysfunctional response to one's own body would predict the activation of a different pattern: inhibition of the eye-blink [21] and potentiation of the first acceleration-deceleration component of the cardiac defense [38]. In addition to these physiological measures, we also recorded the skin conductance response —a well-known index of attentional and emotional arousal [42], [43]— and the subjective reactions of participants towards viewing their own body (ratings of pleasantness, arousal, and control) using the Self-Assessment Manikin [44].

## Method

### Participants

The participants were 60 female students from the University of Granada (Spain), aged 18 to 30 years, who volunteered to take part in a study on body image. They were selected from an initial pool of 1305 students who reported bulimic symptoms and body dissatisfaction using the Bulimic Investigatory Test, Edinburgh (BITE) [45] and the Body Shape Questionnaire (BSQ) [46], respectively. Potential participants scoring above the recommended cut-off for the diagnosis of BN on the two questionnaires (i.e., a BITE total score above 20 and a BSQ score above 105) were provided an individual appointment by telephone to participate in a diagnostic interview conducted by a licensed clinical psychologist. The diagnosis was confirmed through a structured clinical interview based on the DSM-IV [47] diagnostic criteria for BN. The main inclusion criteria for BN were as follows: a) the presence of recurrent binge eating accompanied by loss of control over eating and inappropriate compensatory behaviours; b) duration of binge eating and compensatory behaviours (at least twice per week for three months); and c) a self-evaluation overly influenced by body weight and shape. A similar group of participants scoring below the cut-off for high risk of BN (i.e., a BITE total score below 10 and a BSQ score below 55) were also interviewed by the same clinician to confirm the absence of BN and other psychiatric illnesses. Exclusion criteria for all participants were as follows: a) the presence of substance abuse or addiction; b) the presence of physical illness, such as heart disease or hypertension; c) the presence of uncorrected visual and/or auditory problems; d) have a history of eating disorders or other mental disorders; and e) be undergoing a psychological or psychiatric/pharmacological treatment for eating disorders or other mental disorders. The final participants were 30 women with diagnosis of BN (the BN group) and 30 healthy women (the HC group). The body mass index (BMI) of each participant was calculated using an electronic scale with a stadiometer. The group means for all measures are summarized in Table 1. Significant differences between the two groups were found, as expected, in the two screening questionnaires and in BMI [48], [49]. Due to the presence of artifacts in the recordings, 4 individuals (2 in each group) were excluded from the statistical analysis of the cardiac defense response, and 6 (2 in the BN group and 4 in the HC group) were excluded from the analysis of the skin conductance response. Following their participation, women diagnosed with BN were offered treatment at the University's Psychology Clinic. All participants were given course credit for their participation.

**Table 1:** The general characteristics of participants.

	BN group (n = 30)*	HC group (n = 30)	*t*-Value	*p*-Value
**Age (years)**	20.33 (2.7)	19.8 (2.3)	.814	.419
**Range**	18–30	18–26		
**BMI(kg/m^2^)**	24.48 (3.8)	20.49 (2.5)	4.77	.001
**Range**	15.17–32.76	16.7–28.2		
**BITE**	25.3 (4.7)	2.8 (2.1)	23.8	.001
**BSQ**	137.8 (17.5)	45.9 (6.1)	26.3	.001

Values are means (S.D.).

*The bulimia nervosa (BN) group was composed of 7 women conforming to the BN-purging subtype and 23 women conforming to the BN-non-purging subtype.

Note: BN  =  bulimia nervosa; HC  =  healthy control; BMI  =  body mass index; BITE  =  Bulimic Investigatory Test, Edinburgh; BSQ  =  Body Shape Questionnaire.

### Ethics Statement

This study was approved by the ethics committee of the University of Granada (Spain). All participants provided informed, written consent to participate in the experiment at the beginning of the study.

### Procedure

**Figure 1:**
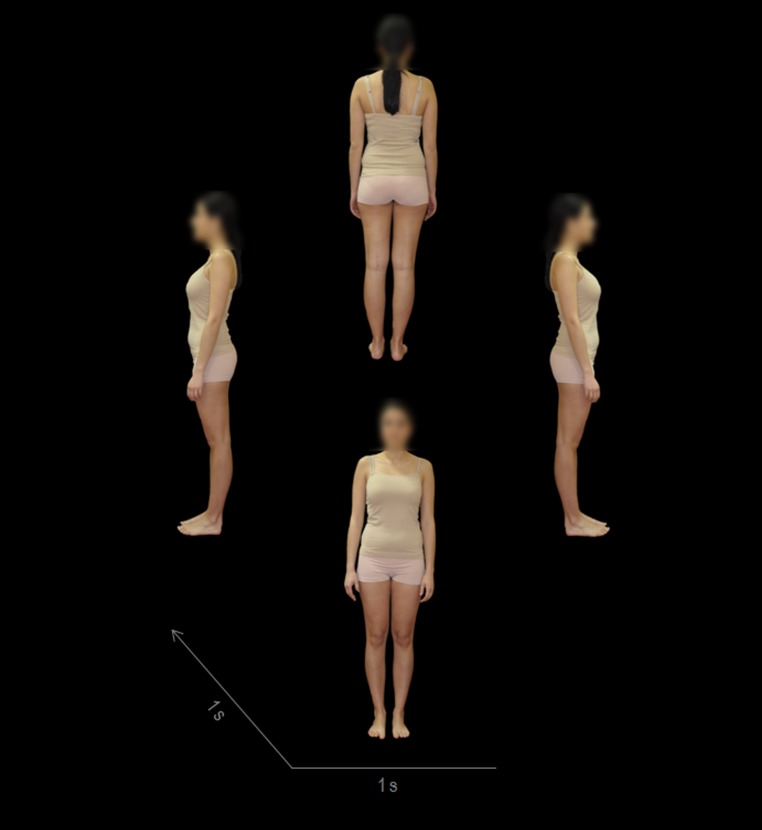
Own body rotating video. The video was created using 4 photographs of each participant in 4 positions (frontal [0°], right profile [90°], back [180°], and left profile [270°]), in this order. The duration of the rotating picture was 9 s (1 s per photograph and 1 s per transition effect, finishing with the frontal photograph maintained during 1 s).

The participants were invited to two laboratory sessions. During the first session, participants received information about the study and provided informed consent for the procedures. Then, a female experimenter in a private room photographed each participant's body after they were instructed to change into nude underpants and a nude tank top. The photographs were taken against a black background, and each participant posed in four positions at 90-degree angles with their arms hanging loosely beside the body (see Fig. 1). All pictures were retouched with Photoshop CS6 software (Adobe Systems, Inc., California, USA) to blur the participants' faces. The decision to blur the participants' faces was taken for three primary reasons. Firstly, face images and body images have proven to be emotionally processed in distinct manners [50]. In fact, face dissatisfaction appears not to be related to body dissatisfaction in women with bulimic symptomatology [51]. Accordingly, it may be difficult to interpret the results obtained by mixing both stimuli, especially when the presentation time is short. Secondly, the ugliest parts of one's own body reported by women with eating disorders are upper legs, hips, belly, and knees, while the ugliest parts reported by normal controls are upper legs, lower legs, hips, and knees [20]. Thus, the participants' bodies, rather than faces, are more appropriate for the assessment of the attentional and emotional impact of one's own body. Finally, it has been demonstrated that people have a tendency to mimic facial expressions [52] and therefore blurring the faces avoids facial processing that could intervene with the interpretation of the results. During the second session, participants underwent a physiological testing according to the startle-defense paradigm. First, the participant was invited to sit in a comfortable chair in a quiet room and was given instructions on the procedure. Next, electrodes were placed to record the eye-blink, the electrocardiogram, and the skin conductance. The physiological testing consisted of a 5-min resting period followed by 2 startle-defense trials. Each trial began with a 15-s baseline recording period followed by a 9-s picture/black presentation and an 80-s post-stimulus recording period. In one trial, the rotating picture composed of the photographs of the participant's own body was presented over the course of 9 s. In the other trial, a black picture simulating the black screen was also presented during a 9-s interval. In both trials, an auditory stimulus —a white noise of 110 dB, 500-ms duration, and instantaneous rise time— capable of eliciting both the eye-blink startle and the cardiac defense response [53] was presented through earphones 8 s after the appearance of the picture/blank. The order of the two trials was counterbalanced within each group to control for the fast habituation of the cardiac defense response. Participants were instructed to view their body's picture for the entire time it was displayed on the screen. They were also told that they would hear brief, intense noises through the earphones that they should ignore. Following each trial, participants rated their subjective feelings of valence, arousal, and control using the Self-Assessment Manikin (SAM) [44]. The second laboratory session was scheduled to start at one of 4 times (10:00 a.m., 12:00 a.m., 4:00 p.m. or 6:00 p.m.) that were equally balanced across the BN and HC groups.

### Apparatus and Physiological Measures

A Biopac MP150 unit connected to a PC-Pentium-4 with AcqKnowledge 4.2 software (Biopac Systems, Inc., Goleta, California) was used to record all physiological variables and stimulus markers at a sampling rate of 1000 Hz.

*Eye-blink* was measured by electromyography (EMG) of the left orbicularis muscle using two miniature Ag/AgCl electrodes with hypertonic electrolyte gel [54]. The electromyographic signal was recorded using an EMG100C Biopac amplifier with 500 gain and a band pass filter with a low cutoff of 10 Hz and a high cutoff of 500 Hz. Later, the signal was filtered offline using a high pass filter of 30 Hz, rectified, and smoothed with a moving average window of 5 sampling points. The startle reflex was defined as the magnitude of the blink response initiated between 21 and 120 ms after the noise onset. The algorithm developed by Globisch et al. [55] was used to establish the magnitude of the blink values, expressed in microvolts. Finally, given the presence of large individual differences in this variable, square root transformations were performed in order to normalize the distribution.

The *electrocardiogram* (ECG) was recorded using two standard-size disposable electrodes with hypertonic electrolyte gel, placed on the right clavicle and left ankle. The ground electrode was placed on the left elbow. The ECG was acquired using an ECG100C Biopac amplifier. The heart-rate response to the startle-defense noise was obtained using the standardized procedure [56]. First, the beat-by-beat heart period (R-R interval) during the 80-s interval following the onset of the noise was transformed into second-by-second heart rate (HR) using weighted averages [57]. These 80 HR values, in beats per minute, were subsequently transformed into difference scores with respect to the mean heart rate recorded during the 15-s baseline period and reduced to 10 values corresponding to the medians of 10 progressively longer intervals: 2 intervals of 3 seconds, 2 intervals of 5 seconds, 3 intervals of 7 seconds, and 3 intervals of 13 seconds. These 10 HR median values corresponded to the pattern of the cardiac defense response: the first acceleration (maximum peak at median 1), the first deceleration (maximum peaks at medians 3 and 4), the second acceleration (maximum peak at median 7), and the second deceleration (maximum peaks at medians 9 and 10). Accordingly, in addition to examine the response pattern of the 10 medians, post-hoc analysis would also test the four cardiac components separately defined as follows: first acceleration: HR value at median 1; first deceleration: first acceleration minus the mean of the HR values at medians 3 and 4; second acceleration: HR value at median 7; and second deceleration: second acceleration minus the mean of the HR values at medians 9 and 10. All individual analyses were performed with the KARDIA software [58].

*Skin conductance* (SC) was recorded by two standard-size Ag/AgCl electrodes, with isotonic electrolyte gel, placed on the middle phalanges of the index and middle fingers of the left hand. Acquisition of this variable was performed with a GSR100C Biopac amplifier. The conductance response to the startle-defense noise was measured in microSiemens, following the same procedure used for the cardiac defense response. The 80 second-by-second skin conductance values following the onset of the noise were transformed into difference scores with respect to the 15-s baseline period and then reduced to 10 median values corresponding to the same 10 intervals used for cardiac defense. This procedure was applied to the skin conductance data for comparative reasons. The response is always a steady increase in conductance beginning between 1 and 4 s after stimulus onset and reaching a peak few seconds later. Afterwards, the response starts decreasing with different decremental rates depending on individual and situational factors. It was expected that the first median value would detect the beginning of the response and the second median value the peak of the response, with the subsequent median values detecting the response's decremental phase.

The *body images* were presented on an LCD monitor placed in the experimental room at a distance of 50 cm from the participant's eyes. Sequence and timing of the body images were controlled through E-Prime 2.0 software (Psychology Software Tools, Inc., Sharpsburg, USA) running on a second PC.

The *startle-defense noise* was presented binaurally through earphones (Sennheiser HD25-1) using a sound generator (Coulbourn V85-05) with an audio amplifier (IMQ Stage Line). The sound intensity was calibrated using a sound meter (Brüel & Kjaer 2235) and an artificial ear (Brüel & Kjaer 4153).

### Self-Report Measures

The *Bulimic Investigatory Test, Edinburgh* (BITE) [45] provides information about eating patterns related to the consumption of food and the practices of binge eating, purging, and dieting. It has two scales. The first scale identifies the presence of bulimic symptoms. The second scale measures the severity of the bulimic symptoms. A total score of 15–20 indicates moderate risk to suffer bulimia nervosa. It shows high internal consistency (Cronbach's alpha: 0.96) and has been adapted to the Spanish population [59].

The *Body Shape Questionnaire* (BSQ) [46] is a 34-item, self-applied questionnaire that assesses concerns regarding body shape. Respondents must indicate the frequency with which they experience body dissatisfaction in cognitive, affective, and behavioral domains.). The clinical cut-off point is ≥ 105 points. In this study we used the adapted Spanish version of the BSQ, which has adequate psychometric properties (Cronbach's alpha: 0.95–0.97) [60].

The *Self-Assessment Manikin* (SAM) [44] consists of 3 scales resembling human-like figures that embody the concepts of valence, arousal, and dominance. Each affective dimension is composed by 9 intensity levels. SAM's valence dimension ranges from very unpleasant (1) to very pleasant (9); the arousal dimension ranges from very calm (1) to very excited (9); and the dominance-control dimension ranges from very dominated/controlled (1) to very dominant/in control (9). This subjective assessment method has been extensively validated and is widely used in cue reactivity research [28], [61].

### Statistical analysis

SAM ratings were analyzed using a 2×2 ANOVA with one between-subjects and one repeated-measures factor. The between-subjects factor was *Group* (i.e., the BN versus the HC group), and the repeated-measures factor was *Trial-Type* (i.e., no picture with sound [Sound Only] versus one's own body picture with sound [Own Body]). The three physiological variables (viz., eye-blink startle, cardiac defense, and skin conductance) were analyzed using a similar design with the addition of a second between-subjects factor (viz., *Trial-Order*, with two levels: Order 1 [the Sound Only trial first and the Own Body trial second] versus Order 2 [the Own Body trial first and the Sound Only trial second]). In the cases of cardiac defense and skin conductance, a second repeated-measures factor, *Medians* (Mdn), was added to represent the response pattern along 10 time-intervals (Mdn1, Mdn2, Mdn3, Mdn4, Mdn5, Mdn6, Mdn7, Mdn8, Mdn9, and Mdn10). In all repeated-measures factors, the Greenhouse-Geisser epsilon correction was applied. Effect sizes of the significant differences are reported as squared partial etas (ηp^2^). When significant interaction effects were found, follow-up analyses were performed on the highest-level interaction in order to identify the factors explaining the effects. Then, when appropriate, post-hoc analyses were performed using Bonferroni-corrected *t*-tests. In the case of cardiac defense, post-hoc analysis on the *Medians* factor also included testing the four cardiac components separately. The level of significance was set at p<.05.

## Results

### SAM ratings

Table 2 shows the means and standard errors of the SAM ratings as a function of *Group* and *Trial-Type*. The results of the 2×2 (*Group* × *Trial-Type*) ANOVA for Valence ratings showed significant effects of *Group,* (*F*(1,58)  = 39.77, p<.0001; ηp^2^ = .407), and *Group* × *Trial-Type* interaction (*F*(1,58)  = 9.29, p<.003; ηp^2^ = .138). Analysis of the interaction revealed that participants in the BN group showed significantly less pleasure during both trial-types than did participants in the HC group (Sound Only trial: *t*(58)  = −3.58, p<.001; Own Body trial: *t*(58)  = −7.08, p<.0001). However, BN participants also showed significantly less pleasure during the Own Body trial than during the Sound Only trial (*t*(29)  = 2.92, p<.01), whereas no *Trial-Type* differences were found in the HC group (p = .24). As shown in Table 2, mean values for BN group were below the midpoint of the valence scale (unpleasant zone), whereas mean values for HC group were above the midpoint of the scale (pleasant zone). The ANOVA results for Arousal ratings showed significant main effects of *Group* (*F*(1,58)  = 12.81, p<.001; ηp^2^ = .181) and *Trial-Type* (*F*(1,58)  = 6.56, p<.013; ηp^2^ = .102). Participants in both groups reacted with more arousal to the Own Body trial than to the Sound Only trial. However, the reaction of the BN group during both trials was significantly larger than the reaction of the HC group. Mean values for BN group were above the midpoint of the arousal scale (high arousal zone), whereas mean values for HC group were below the midpoint of the scale (low arousal zone). Finally, the ANOVA results for Dominance ratings revealed significant effects of *Group* (*F*(1,58)  = 17.26, p<.0001; ηp^2^ = .229), *Trial-Type* (*F*(1,58)  = 4. 91, p<.031; ηp^2^ = .078), and *Group* × *Trial-Type* interaction (*F*(1,58)  = 7.80, p<.007; ηp^2^ = .119). Analysis of the interaction revealed that BN participants felt less control during both trial-types than HC participants (Sound Only trial: *t*(58)  = −2.73, p<.01, and Own Body trial: *t*(58)  = −5.05, p<.001). However, BN participants also felt significantly less control during the Own Body trial than during the Sound Only trial (*t*(29)  = 2.77, p<.01), whereas no *Trial-Type* differences were found in the HC group (p = .5). Mean values for BN group were below the midpoint of the dominance scale (low sense of control), whereas mean values for HC group were above the midpoint of the scale (high sense of control).

**Table 2:** The means (and standard errors of the means) of Valence, Arousal, and Dominance ratings during the Sound Only trial and the Own Body trial.

	VALENCE		AROUSAL		DOMINANCE	
	Trial Type		Trial Type		Trial Type	
	*Sound Only*	*Own Body*	*Sound Only*	*Own Body*	*Sound Only*	*Own Body*
**BN**	4.9 (0.3)	3.9 (0.3)	5.6 (0.4)	6.4 (0.4)	4.4 (0.3)	3.6 (0.3)
**HC**	6.3 (0.3)	6.6 (0.3)	4.1 (0.4)	4.3 (0.4)	5.7 (0.3)	5.8 (0.3)

Note: BN  =  bulimia nervosa group; HC  =  healthy control group.

### Startle reflex

**Figure 2:**
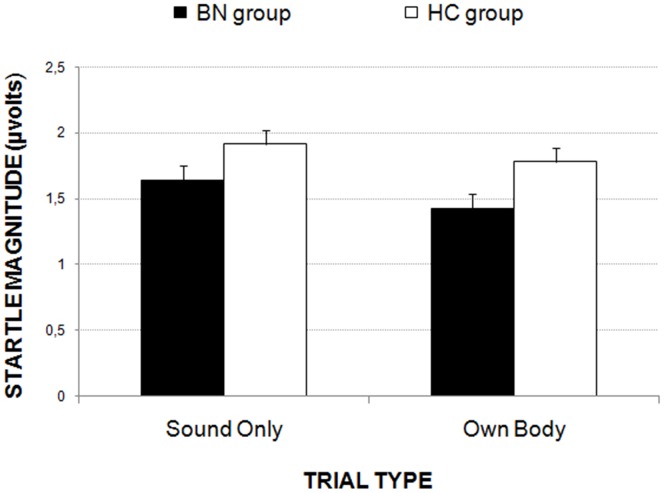
The eye-blink reflex magnitude to the startle-defense noise during the Sound Only and Own Body trials for both groups of participants.

The results of the 2×2×2 ANOVA (*Group* × *Trial-Order* × *Trial-Type*) showed two significant main effects: *Group* (*F*(1,56)  = 4.59, p<.037; ηp^2^ = .076) and *Trial-Type* (*F*(1,56)  = 11.66, p<.001; ηp^2^ = .172). As shown in Fig. 2, both groups exhibited smaller blink magnitudes in the Own Body trial than in the Sound Only trial. In addition, eye-blink magnitude was significantly smaller for women with BN compared with the HC women.

### Cardiac defense response

**Figure 3:**
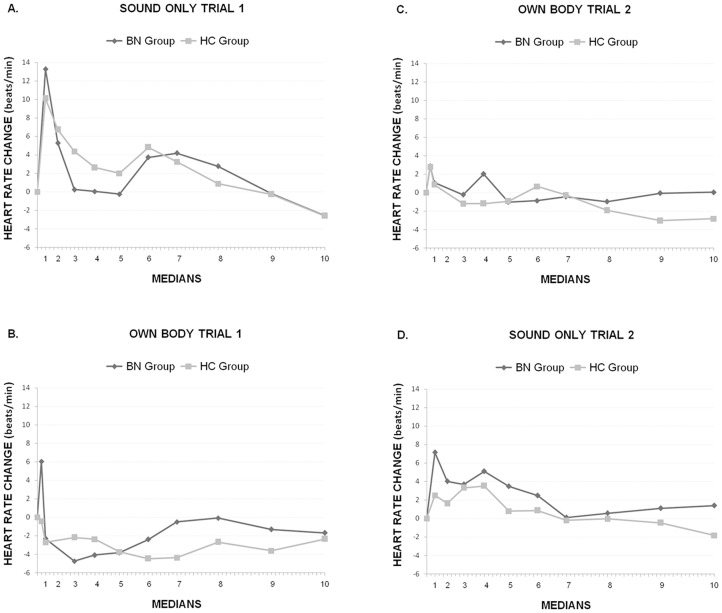
The heart-rate response to the startle-defense noise in the Bulimia (BN) and Control (CN) groups during the Sound Only trial (A) and the Own Body trial (B) presented first, and the Own Body trial (C) and the Sound Only trial (D) presented second. The typical cardiac defense response pattern was observed only in the response to the Sound Only trial presented first (A). In the Own Body trial presented first, the healthy control (HC) group showed no response, whereas the bulimia nervosa (BN) group showed the initial acceleration-deceleration pattern followed by a return to baseline (B). When both trials were presented second, the response pattern did not appear (C and D).

The 2×2×2×10 ANOVA (*Group* × *Trial-Order* × *Trial-Type* × *Medians*) revealed significant effects of *Trial-Type* (*F*(1,51)  = 17.52, p<.0001; ηp^2^ = .256), *Medians* (*F*(9,459)  = 13.78, p<.0001; ηp^2^ = .213), *Trial-Type* × *Medians × Trial-Order* (*F*(9,459)  = 6.13, p<.0001; ηp^2^ = .107) and *Trial-Type* × *Medians × Trial-Order* × *Group* (*F*(9,459) = 2.82, p<.018; ηp^2^ = .052). Fig. 3 illustrates the four-way interaction. The typical response pattern, with its two acceleration-deceleration components, was observed in both groups in response to the Sound Only trial presented first (Fig. 3A). A similar biphasic response pattern, with a large first acceleration-deceleration and an attenuated second acceleration-deceleration, was also observed in response to the Own Body trial presented first, but only in the BN group (Fig. 3B). The HC group showed a single prolonged deceleration in this trial. Finally, when the Sound Only trial and the Own Body trial were presented second (Fig. 3C and 3D, respectively), the typical cardiac defense response pattern did not appear. Follow-up analysis of the four-way interaction revealed a significant *Medians* × *Trial-Order* × *Group* only in the Own Body trial (*F*(9,459)  = 3.02, p<.014; ηp^2^ = .056). In the Sound Only trial this interaction was not significant (p = .5). Further, significant *Medians* × *Group* interaction appeared when the Own Body trial was presented first (*F*(9,207)  = 2.60, p<.046; ηp^2^ = .102). When the Own Body trial was presented second, this interaction was not significant (p = .18). Finally, only the BN group showed a significant *Medians* effect when the Own Body trial was presented first (*F*(9,117)  = 5.70, p<.002; ηp^2^ = .305). No *Medians* effect was found in the control group (p<.16). In addition, post-hoc analysis to test group differences in the four cardiac defense components when the Own Body trial was presented first, revealed significant differences in the first acceleration (*t*(24)  = 2.27, p<.032) and first deceleration (*t*(24)  = 2.39, p<.025). In both cases, the BN showed a larger first acceleration and a larger first deceleration than the HC group. In the second acceleration (p = .162) and second deceleration (p = .07) the group differences were not significant.

### Skin Conductance Response

**Figure 4:**
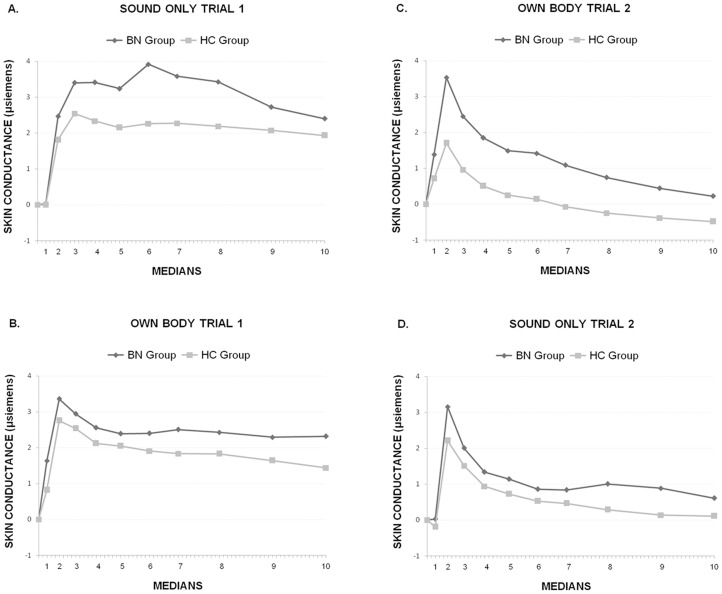
The skin conductance response to the startle-defense noise in the Bulimia (BN) and Control (CN) groups during the Sound Only trial (A) and the Own Body trial (B) presented first, and the Own Body trial (C) and the Sound Only trial (D) presented second. In general, the BN group showed a larger response to both trial types, irrespective of the presentation order. Also, when both trials were presented second (C and D), the amplitude of the response decreased significantly after Median 2 in the BN and HC groups, suggesting a habituation effect. However, at the start of this second presentation (Median 1), the response was larger for the Own Body trial than for the Sound Only trial, and for the BN group compared to the HC group, indicating less habituation.

The 2×2×2×10 ANOVA (*Group* × *Trial-Order* × *Trial-Type* × *Medians*) revealed significant effects of *Group* (*F*(1,50)  = 4.20, p<.046; ηp^2^ = .077), *Medians* (*F*(9,450)  = 28.05, p<.0001, ηp^2^ = .359), *Trial-Type* × *Medians* (*F*(9,450)  = 7.12, p<.002; ηp^2^ = .125), and *Trial-Type* × *Medians × Trial-Order* (*F*(9,450)  = 14.57, p<.0001; ηp^2^ = .226). Fig. 4 illustrates these results. In general, the BN group showed a larger response in both the Sound Only trial (Fig. 4A and 4D) and the Own Body trial (Fig. 4B and 4C) relative to the HC group, as reflected in the main effect of *Group*. There was also an observable reduction in the amplitude of the response, after Median 2, in the second presentation for both trial types, as reflected by the three-way interaction. This reduction can be understood in terms of habituation tendency. Follow-up analysis of the *Trial-Type* × *Medians Trial-Order* interaction revealed significant *Trial-Type* × *Medians* interaction both when the Own Body trial was presented first followed by the Sound Only trial (*F*(9,225)  = 4.00, p<.02; ηp^2^ = .138) and when the Sound Only trial was presented first followed by the Own Body trial (*F*(9,225)  = 12.51, p<.0001; ηp^2^ = .333). In both cases, the significant reduction occurred in the second presentation, irrespective of Trial-Type. However, this reduction was observed only after Median 2 (all ps <.02). In Median 1, the response was significantly larger for the Own Body trial than for the Sound Only trial, irrespective of trial order (*F*(1,50)  = 39.62, p<.0001; ηp^2^ = .442) and, reflecting the general *Group* effect, larger for the BN group than the HC group (*F*(1,50)  = 6.96, p<.01; ηp^2^ = .122).

## Discussion

In this study we used the startle-defense response paradigm to investigate the relative importance of emotional and attentional dysfunctional processes activated in women with BN during the visualization of their own body. The data from the subjective reports indicate that, in the context of the startle-defense paradigm, women with BN, compared to healthy women, perceive their own bodies as evoking more unpleasant feelings and less sense of control during the Own Body trial than during the Sound only trial. No differences between either trial types were found in healthy women, who reported more pleasant feelings and more sense of control than BN women. Both groups reported higher subjective arousal during the Own Body trial than during the Sound Only trial, but the arousal ratings of women with BN were significantly higher than those of healthy women. Skin conductance results –a measure of sympathetic emotional activation– further support the differences in subjective arousal observed between BN patients and healthy women. The BN group showed, in general, a larger skin conductance response than the HC group, irrespective of trial-type. However, when the Own Body trial was presented second, the response was also larger at the beginning of the response (Median 1), indicating a reduced habituation effect in the BN patients compared to the controls. Taken together, subjective reports and skin conductance results confirm that one's own body is perceived by BN patients as an unpleasant, uncontrollable, and activating stimulus.

Emotional activation alone, however, is not sufficient for explaining our findings on the startle-defense responses elicited by one's own body. Contrary to the expected pattern of startle potentiation that has been consistently reported when people view highly unpleasant stimuli, we found that the startle response was inhibited when our participants viewed videos of their own body. A similar startle inhibition in response to self-images was previously reported by Buck et al. [21]. Our study supports this finding and extends it to women with BN, showing for the first time that these patients experience greater startle inhibition to self-images, probably indicating increased attentional engagement compared to healthy women.

In addition, our BN patients demonstrated a smaller startle response than that of healthy women in the Sound Only trial, when there was no visualization of the body. This startle inhibition, although of less magnitude than that observed during visualization of the body, might be explained as reflecting a generalized tonic state of alertness during the entire task. Alertness is conceptualized as a state of general wakefulness that facilitates a high level of responsiveness in anticipation to an expected event [62], [63]. It is accompanied by both, an excitability component, indexed by an increase in sympathetic nervous system activity, and an inhibitory motor component, indexed by a marked reduction in irrelevant movements including a steady, unblinking eye [64], [65]. A state of general alertness might therefore be responsible for the attenuated startle response in BN participants during the Sound Only trial, as they were aware that the task to follow involved observing images of their own body. Our subjective reports, indicating that BN women were in a more negative emotional state during the Sound Only trial, compared to the HC group, are consistent with this interpretation.

The cardiac defense response data also supports the attentional predominance of the BN women's response during the visualization of self-images. Thus, in the BN group we found a potentiation of the first acceleration-deceleration, whereas the control group exhibited a general inhibition of the response. This different response pattern between BN and HC participants was observed only when the own body was presented in the first trial, probably due to the fast habituation of the cardiac defense response [25], [37]. It is well established that the first cardiac component, mediated by parasympathetic control, is related to the attentional phase of the defensive response: interruption of ongoing activity and increased attention to potentially threatening stimuli [25], [38]. The significant attentional engagement of BN women is further corroborated by the dynamical pattern of the whole cardiac response: initial heart-rate acceleration followed by a steep deceleration before returning to baseline (without the second acceleration). This response pattern has been interpreted as reflecting a passive defensive style characterized by the use of defensive strategies such as freezing, a natural fear-induced immobility response to threatening situations [25].

Evidence from animal models indicates that aversive stimulation may cause defensive behaviors in a hierarchical manner: alertness, freezing, and flight-fight responses [66]. Similarly, a defense cascade model has been proposed to explain defensive reactions in humans [30]. According to the defense cascade model, defensive reactions are characterized by three consecutive phases: pre-encounter, post-encounter, and circa-strike, which ranges from attentive freezing to active defense depending on the proximity of the threatening stimulus. In line with this perspective, we may interpret our findings as indicating that the mere anticipation of self-body viewing is capable of activating the initial alertness stage of the defense cascade in BN women: attenuated startle response in the Sound Only trial compared to the healthy group. Importantly, the actual presentation of the aversive stimulus (own body) seems to advance the defense cascade to its next stage of behavioral freezing, indexed by startle inhibition and a passive-coping cardiac defense pattern. On the other hand, the topography of the cardiac defense response in the control group, showing an absence of the two typical acceleration-deceleration components, implies that in healthy women viewing one's own body does not evoke a defensive reaction. Instead, self-body perception seems to protect against the capacity of the aversive stimuli (i.e., the loud noise) to activate defense reactions, as indexed by an attenuated overall cardiac defense response.

In conclusion, our results clearly indicate that the methodology used in the present research is appropriate for the assessment of the emotional and attentional mechanisms underlying body dissatisfaction. Furthermore, this is the first study to employ the cardiac defense response in the context of body image disturbances. Despite the importance of body dissatisfaction as a risk factor to relapse in BN [4], objective measures may benefit the assessment of this clinical condition. Our study advances previous research focused on body image and highlights the prevalence of attentional mechanisms in the physiological response of women with BN to images of their own body. This finding has two relevant clinical implications. First, the startle-defense paradigm might be a useful assessment tool for both: i) the prediction/diagnosis of body image disturbances in BN patients, and ii) the efficacy of therapeutic interventions aimed at reducing body dissatisfaction. Second, our results show that self-images captivate the attention of BN patients and that this attentional engagement is fundamental in the generation of dysfunctional physiological responses. Thus, therapeutic interventions utilizing body exposure to attenuate aversive emotions induced by one's own body [6]–[10] could benefit by the implementation of strategies aimed to reduce attentional biases, as also suggested by eye-tracker studies [20], [67].

## References

[1] Stice E, Shaw HA (2002) Role of body dissatisfaction in the onset and maintenance of eating pathology: A synthesis of research findings. J Psychosom Res 53: 985–993. 12445588 10.1016/s0022-3999(02)00488-9

[2] Viken RJ, Treat TA, Nosofsky RM, McFall RM, Palmeri TJ (2002) Modeling individual differences in perceptual and attentional processes related to bulimic symptoms. J Abnorm Psychol 111: 598–609 10.1037/0021-843X.111.4.598 12428773

[3] Stice E (2002) Risk and maintenance factors for eating pathology: A meta-analytic review. Psychol Bull 128: : 825-848. doi: 10.1037/0033–2909.128.5.825 10.1037/0033-2909.128.5.825 12206196

[4] Cash T, Hrabosky JI (2004) Treatment of body image disturbances. In: Thompson JK, editor.Handbook of eating disorders and obesity.New York: Wiley. pp. 515–541.

[5] Rosen JC (1996) Body image assessment and treatment in controlled studies of eating disorders. Int J Eat Disord 20: 331–343 doi: ;10.1002/(SICI)1098-108X(199612)20: 4<331::AID-EAT1>3.0.CO;2-O 8953321 10.1002/(SICI)1098-108X(199612)20:4<331::AID-EAT1>3.0.CO;2-O

[6] Tuschen-Caffier B, Pook M, Frank M (2001) Evaluation of manual-based cognitive-behavioral therapy for bulimia nervosa in a service setting. Behav Res Ther 39: 299–308 10.1016/S0005-7967(00)00004-8 11227811

[7] Norris DL (1984) The effects of mirror confrontation on self-estimation of body dimensions in anorexia nervosa, bulimia and two control groups. Psychol Med 14: 835–842 10.1017/S0033291700019802 6599508

[8] Delinsky SS, Wilson GT (2010) Cognitive behavior therapy with body image exposure for bulimia nervosa: A case example. Cogn Behav Pract 17: 270–277 10.1016/j.cbpra.2010.02.004

[9] Moreno-Domínguez S, Rodríguez-Ruiz S, Fernández-Santaella MC, Jansen A, Tuschen-Caffier B (2012) Pure versus guided mirror exposure to reduce body dissatisfaction: A preliminary study with university women. Body image 9: 285–288 10.1016/j.bodyim.2011.12.001 22244836

[10] Trentowska M, Bender C, Tuschen-Caffier B (2013) Mirror exposure in women with bulimic symptoms: How do thoughts and emotions change in a body image treatment? Behav Res Ther 51: 1–6 10.1016/j.brat.2012.03.012 23168326

[11] Cooper MJ, Fairburn CG (1992) Thoughts about eating, weight and shape in anorexia nervosa and bulimia nervosa. Behav Res Ther 30: 501–511 10.1016/0005-7967(92)90034-E 1520236

[12] Tuschen-Caffier B, Vögele C, Bracht S, Hilbert A (2003) Psychological responses to body shape exposure in patients with bulimia nervosa. Behav Res Ther 41: 573–586 10.1016/S0005-7967(02)00030-X 12711265

[13] Vocks S, Legenbauer T, Wächter A, Wucherer M, Kosfelder J (2007) What happens in the course of body exposure? Emotional, cognitive, and physiological reactions to mirror confrontation in eating disorders. J Psychosom Res 62: 231–239. 17270582 10.1016/j.jpsychores.2006.08.007

[14] Laberg JC, Wilson GT, Eldredge K, Nordby H (1991) Effects of mood on heart rate reactivity in bulimia nervosa. Int J Eat Disord 10: 169–178 doi: ;10.1002/1098-108X(199103)10: 2<169::AID-EAT2260100205>3.0.CO;2-Y

[15] Overduin J, Jansen A, Eilkes H (1997) Cue reactivity to food- and body-related stimuli in restrained and unrestrained eaters. Addict Behav 22: 395–404 10.1016/S0306-4603(97)80002-0 9183509

[16] Beato-Fernández L, Rodríguez-Cano T, García-Vilches I, García-Vicente A, Poblete-García V, et al (2009) Changes in regional cerebral blood flow after body image exposure in eating disorders. Psychiat Res-Neuroim 171: 129–137 10.1016/j.pscychresns.2008.01.001 19168335

[17] Reas DL, Whisenhunt BL, Netemeyer R, Williamson DA (2002) Development of the body checking questionnaire: A self-report measure of body checking behaviors. Int J Eat Disord 31: 324–333 10.1002/eat.10012 11920995

[18] Smeets E, Roefs A, Van Furth E, Jansen A (2008) Attentional bias for body and food in eating disorders: Increased distraction, speeded detection, or both? Behav Res Ther 46: 229–238 10.1016/j.brat.2007.12.003 18191812

[19] Smeets E, Tiggemann M, Kemps E, Mills JS, Hollitt S, et al (2011) Body checking induces an attentional bias for body-related cues. Int J Eat Disord 44: 50–57 10.1002/eat.20776 19950112

[20] Jansen A, Neederkorn C, Mulkens S (2005) Selective visual attention for ugly and beautiful body parts in eating disorders. Behav Res Ther 43: 183–196 10.1016/j.brat.2004.01.003 15629749

[21] Buck SM, Hillman CH, Evans EM, Janelle CM (2004) Emotional responses to pictures of oneself in healthy college age females. Motiv Emot 28: 279–295 10.1023/B:MOEM.0000040155.79452.23

[22] Fairburn CG, Peveler RC, Jones R, Hope RA, Doll HA (1993) Predictors of 12-month outcome in bulimia nervosa and the influence of attitudes to shape and weight. J Consult Clin Psychol 61: 696–698 10.1037/0022-006X.61.4.696 8370866

[23] Fairburn CG, Stice E, Cooper Z, Doll HA, Norman PA, et al (2003) Understanding persistence in bulimia nervosa: A 5-year naturalistic study. J Consult Clin Psychol 71: 103–109 10.1037/0022-006X.71.1.103 12602430

[24] Lang PJ (1995) The emotion probe. Studies of motivation and attention. Am Psychol 5: 372–385 10.1037/0003-066X.50.5.372 7762889

[25] Vila J, Guerra P, Muñoz MA, Vico C, Viedma-del Jesús MI, et al (2007) Cardiac defense: From attention to action. Int J Psychophysiol 66: 169–182 10.1016/j.ijpsycho.2007.07.004 17706311

[26] Strauss H (1929) Das zusammenschrecken: Experimentell-Kinematographische Studie zur Physiologie und Pathophysiologie der Reaktivbewegungen. J Psychol Neurol 39: 111–231.

[27] Graziano MSA, Cooke DF (2006) Parieto-frontal interactions, personal space, and defensive behavior. Neuropsychologia 44: 845–859 10.1016/j.neuropsychologia.2005.09.009 16277998

[28] Bradley MM, Lang PJ (2007) The International Affective Picture System (IAPS) in the study of emotion and attention. In: Coan JA, Allen JJB, editors.Handbook of Emotion Elicitation and Assessment.New York: Cambridge University Press. pp. 29–46.

[29] Davis M (1992) The role of amygdala in fear potentiated startle: Implications for animal models of anxiety. Trends Pharmacol Sci 13: 35–41 10.1016/0165-6147(92)90014-W 1542936

[30] Lang PJ, Davis M, Öhman A (2000) Fear and anxiety: Animal models and human cognitive psychophysiology. J Affect Disord 61: 137–159 10.1016/S0165-0327(00)00343-8 11163418

[31] Yeomans JS, Frankland PW (1996) The acoustic startle reflex: Neurons and connections. Brain Res Rev 21: 301–314 10.1016/0165-0173(96)00004-5 8806018

[32] Hoebel BG, Rada PV, Mark GP, Pothos EN (1999) Neural systems for reinforcement and inhibition of behavior: Relevance to eating, addiction, and depression. In: Kahneman D, Diener E, Schwarz N, editors.Well-being: The foundations of hedonic psychology.New York: Russell Sage Foundation. pp. 558–572.

[33] Filion DL, Dawson ME, Schell AM (1998) The psychological significance of human startle eyeblink modification: A review. Biol Psychol 47: 1–43 10.1016/S0301-0511(97)00020-3 9505132

[34] Lang PJ, Bradley MM, Cuthbert BN (1997) Motivated attention: Affect, activation, and action. In: Lang PJ, Simons RF, Balaban MT, editors.Attention and Orienting: Sensory and Motivational processes.Mahwah: Lawrence Erlbaum Associates. pp. 97–135.

[35] Lipp OV, Hardwick SA (2003) Attentional blink modulation in a reaction time task: Performance feedback, warning stimulus modality, and task difficulty. Biol Psychol 62: 115–132 10.1016/S0301-0511(02)00115-1 12581687

[36] Bond DD (1943) Sympathetic and vagal interaction in emotional responses of the heart rate. Am J Physiol 138: 468–478.

[37] Mata JL, Rodríguez-Ruiz S, Ruiz-Padial E, Turpin G, Vila J (2009) Habituation and sensitization of protective reflexes: Dissociation between cardiac defense and eye-blink startle. Biol Psychol 81: 192–199 10.1016/j.biopsycho.2009.04.006 19397949

[38] Keil A, Bradley MM, Ihssen N, Heim S, Vila V, et al (2010) Defensive engagement and perceptual enhancement. Neuropsychologia 48: 3580–3584 10.1016/j.neuropsychologia.2010.08.007 20713075 PMC2949445

[39] Ramírez I, Guerra P, Muñoz MA, Perakakis P, Anllo-Vento L, et al (2010) The dynamics of cardiac defense: From attention to action. Psychophysiol 47: 879–887 10.1111/j.1469-8986.2010.01008.x 20374542

[40] McTeague LM, Lang PJ, Laplante MC, Cuthbert BN, Shumen JR, et al (2010) Aversive imagery in posttraumatic stress disorder: trauma recurrence, comorbidity, and physiological reactivity. Biol Psychiatry 67: 346–356 10.1016/j.biopsych.2009.08.023 19875104 PMC3747632

[41] Ruiz-Padial E, Vila J (2007) Fearful and sexual pictures not consciously seen modulate the startle reflex in human beings. Biol Psychiatry 61: 996–1001 10.1016/j.biopsych.2006.08.046 17161828

[42] Dawson ME, Schell AM, Filion DL (2007) The electrodermal system. In: Cacioppo JT, Tassinary LG, Berntson G, editors.Handbook of psychophysiology.New York: Cambridge University Press. pp. 159–181.

[43] Lang PJ, Greenwald MK, Bradley MM, Hamm AO (1993) Looking at pictures: Affective, facial, visceral, and behavioral reactions. Psychophysiol 30: 261–273 10.1111/j.1469-8986.1993.tb03352.x 8497555

[44] Lang PJ (1980) Behavioral treatment and bio-behavioral assessment: Computer applications. In: Sidowski JB, Johnson JH, Williams TA, editors.Technology in mental health care delivery systems.Norwood: Ablex. pp. 119–137.

[45] Henderson M, Freeman CCL (1987) A self-rating scale for bulimia: The BITE. Brit J Psychiat 150: 18–24 10.1192/bjp.150.1.18 3651670

[46] Cooper MJ, Taylor MJ, Cooper Z, Fairburn CG (1987) The development and validation of the body shape questionnaire. Int J Eat Disord 6: 485–494 doi: ;10.1002/1098-108X(198707)6: 4<485::AID-EAT2260060405>3.0.CO;2-O

[47] American Psychiatric Association (2000) Diagnostic and Statistical Manual of Mental Disorders-IV Text Revision. Washington: American Psychiatric Press.

[48] Carlson JD (2004) Body image among adolescent girls and boys: A longitudinal study. Dev Psychol 40: 823–835 10.1037/0012-1649.40.5.823 15355169

[49] Paxton SJ, Eisenberg ME, Neumark-Sztainer D (2006) Prospective predictors of body dissatisfaction in adolescent girls and boys: A five year longitudinal study. Dev Psychol 42: 888–899 10.1037/0012-1649.42.5.888 16953694

[50] Slaughter V, Stone V, Reed C (2004) Perception of faces and bodies: Similar or different? Curr Dir Psychol Sci 13: 219–223 10.1111/j.0963-7214.2004.00312.x

[51] Maner JK, Holm-Denoma JM, Van Orden KA, Gailliot MT, Gordon KH, et al (2006) Evidence for attentional bias in women exhibiting bulimotypic symptoms. Int J Eat Disord 39: 55–61 10.1002/eat.20222 16231350

[52] Dimberg U, Thunberg M, Elmehed K (2000) Unconscious facial reactions to emotional facial expressions. Psych Sci 11: 86–89 10.1111/1467-9280.00221 11228851

[53] Ramírez I, Sánchez MB, Fernández MC, Lipp OV, Vila J (2005) Differentiation between protective reflexes: Cardiac defense and startle. Psychophysiol 42: 732–739 10.1111/j.1469-8986.2005.00362.x 16364069

[54] Fridlund AJ, Cacioppo JT (1986) Guidelines for human electromyographic research. Psychophysiol 23: 567–589 10.1111/j.1469-8986.1986.tb00676.x 3809364

[55] Globisch J, Hamm A, Schneider R, Vaitl D (1993) A computer program for scoring reflex eyeblink and electrodermal responses written in Pascal. Psychophysiol 39: S30.

[56] Vila J, Fernández MC, Godoy J (1992) The cardiac defense response in humans: effects of stimulus modality and gender differences. J Psychophysiol 6: 140–154.

[57] Reyes del Paso GA, Vila J (1998) The continuing problem of incorrect heart rate estimation in psychological studies: An off-line solution for cardiotachometer users. Biol Psychol 48: 269–279 10.1016/S0301-0511(98)00039-8 9788764

[58] Perakakis P, Joffily M, Taylor M, Guerra P, Vila J (2010) KARDIA: A Matlab software for the analysis of cardiac interbeat intervals. Comput Methods Programs Biomed 98: 83–89 10.1016/j.cmpb.2009.10.002 19896748

[59] Rivas T, Bersabé R, Jiménez M (2004) Reliability and validity of the Bulimic Investigatory Test Edinburgh (BITE) in a sample of Spanish adolescents. Behav Psychol 12: 447–461.

[60] Raich R, Mora M, Soler A, Avila C, Clos I, et al (1996) Adaptation of an instrument to evaluate body dissatisfaction. Clinical and Health 7: 51–66.

[61] Coffey SF, Saladin ME, Drobes D, Brady KT, Dansky BS, et al (2002) Trauma and substance cue reactivity in individuals with comorbid posttraumatic stress disorder and cocaine or alcohol dependence. Drug Alcohol Depen 65: 115–127 10.1016/S0376-8716(01)00157-0 11772473

[62] Posner MI (1975) Psychobiology of attention. In: Gazzaniga M, Blakemore C, editors.Handbook of psychobiology.New York: Academic Press. pp. 441–480.

[63] Van Zomeren AH, Brouwer WH (1994) Clinical neuropsychology of attention. New York: Oxford University Press. 41 p.

[64] Posner MI (1995) Attention in cognitive neuroscience: An overview. In: Gazzaniga M, editor.The cognitive neurosciences.Cambridge: MIT Press. pp. 615–624.

[65] Webb RA, Obrist PS (1970) The physiological concomitants of reaction time performance as a function of preparatory interval and preparatory interval series. Psychophysiol 6: 389–403 10.1111/j.1469-8986 5418807

[66] Lamprea MR, Cardenas FP, Vianna DM, Castilho VM, Cruz-Morales SE, et al (2002) The distribution of fos immunoreactivity in rat brain following freezing and escape responses elicited by electrical stimulation of the inferior colliculus. Brain Res 950: 186–194 10.1016/S0006-8993(02)03036-6 12231243

[67] Jansen A, Smeet T, Artijn C, Nederkoorn C (2006) I see what you see: The lack of a self-serving body image bias in eating disorders. Br J Clin Psychol 45: 123–135 10.1348/014466505X50167 16480571

